# Restoration of Hip Biomechanics after a Hemiarthroplasty for a Femoral Neck Fracture—Who Does It Better?

**DOI:** 10.3390/life12010049

**Published:** 2021-12-29

**Authors:** Dylan Tanzer, Aslan Baradaran, Adam Hart, Michael Tanzer

**Affiliations:** 1 Department of Surgery, University at Buffalo, Buffalo, NY 14215, USA; tanzerd@gmail.com; 2 Division of Orthopaedic Surgery, McGill University, Montreal, QC H3A 0G4, Canada; aslan.baradaran@mail.mcgill.ca (A.B.); adam.hart@mcgill.ca (A.H.)

**Keywords:** hip fracture, biomechanics, offset, leg length, arthroplasty

## Abstract

Introduction: The restoration of the preoperative biomechanics of the hip, in particular leg length and femoral offset, are critical in restoring normal function and diminishing the risk of dislocation following hip arthroplasty. This study compares the consistency of arthroplasty and non-arthroplasty orthopedic surgeons in restoring the normal biomechanics of the hip when performing a hemiarthroplasty for the treatment of a femoral neck fracture. Methods: We retrospectively reviewed the preoperative and postoperative digital radiographs of 175 hips that had a modular hemiarthroplasty for the treatment of a displaced femoral neck fracture at a Level 1 academic hospital. Fifty-two hips were treated by one of the three fellowship-trained arthroplasty surgeons (Group A), and 123 were treated by one of the nine non-arthroplasty fellowship-trained orthopedic surgeons (Group B). Results: Patients in Group A were more likely to have their femoral offset restored to normal than patients in Group B, both with respect to under correcting the offset (*p* = 0.031) and overcorrecting the offset (*p* = 0.010). Overall, there was no difference in restoration of leg lengths between the two groups (*p* = 0.869). Conclusions: Following a hemiarthroplasty for a displaced femoral neck fracture, the normal biomechanics of the hip are more likely to be restored by an arthroplasty-trained surgeon than by a non-arthroplasty-trained surgeon. Identifying the inconsistency of non-arthroplasty surgeons and, to a lesser degree, arthroplasty surgeons in restoring hip biomechanics is important for sensitizing surgeons to rectify this in the future with appropriate templating and femoral implant selection.

## 1. Introduction

Worldwide, there are 1.3 million hip fractures every year, and with our aging population, this is projected to rise to more than 6 million fractures by the year 2050 [1,2,3]. Many types of arthroplasties are utilized in treating displaced fractures of the femoral neck (NOF) in the elderly, including hemiarthroplasty (HA). Despite the recent increase in the use of total hip arthroplasty to treat these hip fractures, HA remains the treatment in the vast majority of patients [4,5].

Reestablishment of the patient’s leg lengths and femoral offset are necessary following hemiarthroplasty in order to restore hip biomechanics [6,7,8,9,10]. The intraoperative restoration of the patient’s normal hip biomechanics is imperative to minimize the risk of dislocation and improve outcomes [10,11]. Shortening of the leg is associated with patient dissatisfaction, muscle weakness and dislocation [6,12,13]. On the other hand, excessive lengthening of the leg can result in imbalance while standing, chondral damage of the acetabulum and aseptic loosening of the stem [6,12,13]. Similarly, a loss of femoral offset results in shortening of the lever arm of the abductors with resultant abductor muscle weakness, limp and the risk of dislocation [6]. Excessive femoral offset can result in lateralization of the greater trochanter into the iliotibial band with resultant bursitis/tendonitis.

Preoperative planning and intraoperative restoration of the native hip biomechanics following hip arthroplasty is routine for orthopedic surgeons who specialize in arthroplasty [9,14]. However, patients with hip fractures having an HA are frequently treated by non-arthroplasty orthopedic surgeons who may be less aware of the importance of restoring the native femoral offset and leg lengths and/or less fastidious in attempting to achieve this intraoperatively. Precise anatomical restoration of the hip can be demanding at times, requiring preoperative planning to choose a femoral implant with an appropriate neck offset and head length, as well as to determine the final location of the hip center after reconstruction [15,16].

Few studies have specifically looked at surgeon expertise or specialization regarding patient outcomes after HA for a femoral neck fracture [17,18,19,20], and only one smaller study has investigated the mean leg length discrepancy and femoral offset difference between arthroplasty and non-arthroplasty surgeons [20]. This study was conducted to determine if there was a difference in the degree of restoration of the native hip biomechanics after HA for a femoral neck fracture performed by arthroplasty and non-arthroplasty surgeons and if this difference was more pronounced within a subspecialty.

## 2. Materials and Methods

After obtaining ethics approval from our institutional review board, we retrospectively reviewed the radiographs of 242 consecutive patients who had undergone a primary modular unipolar or bipolar HA for a traumatic femoral neck fracture between 1 January 2013 and 31 December 2018 at a Level 1 academic trauma center. Patients were excluded if we were unable to determine the native offset of the contralateral femur due to poor quality radiographs, if the postoperative radiographs were not available or if the patient had previous hip surgery on the contralateral side. In addition, patients that were non-ambulatory or had an HA for a pathologic fracture, conversion of a failed internal fixation implant or had a proximal femoral deformity were excluded. The absence of a previous deformity or prior surgery of the fractured proximal femur decreased the likelihood that any preoperative leg length discrepancy resulted in the fall predisposing the patient to incomplete restoration of the leg lengths postoperatively. After these exclusions, there were 175 hips in 175 patients for analysis in this study.

For each patient, the pre- and postoperative anteroposterior (AP) pelvis and AP hip digital radiographs (InteleViewer, Diagnostic Viewer, Intelerad Medical Systems, Montreal, Quebec) were assessed to determine if the surgery restored the patient’s leg lengths and femoral offset. All radiographs were taken with a single metal calibration spherical ball or a pelvic calibration device (KingMark, Brainlab AG, Munich, Germany) in order to eliminate the variable of the X-ray magnification and to accurately measure the desired parameters. Postoperatively, the known unipolar head diameter was also used to determine the magnification of the radiographs.

**Figure 1:**
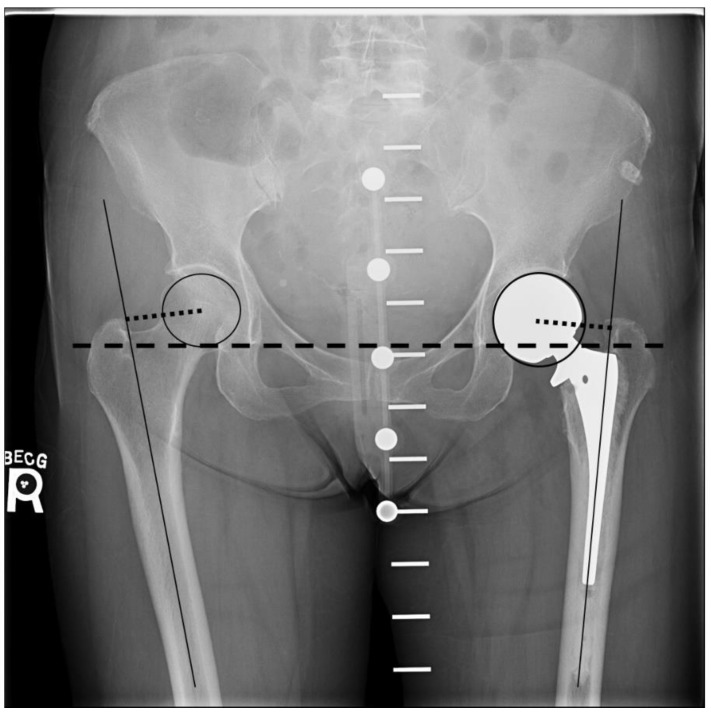
Postoperative AP radiograph of the pelvis with the pelvic calibration device demonstrating a left cemented hemiarthroplasty. The dotted line from the center of the femoral head to the line bisecting the long axis of the femur is the femoral offset. Length discrepancy is determined by measuring the difference in the perpendicular distance from the inter-teardrop line (dashed line) to the superior aspect of the lesser trochanter of each hip.

Measurements to calculate leg lengths and femoral offset were made using TraumaCad digital software (Brainlab AG, Munich, Germany). Postoperatively, a leg length discrepancy between the operated leg and the contralateral leg was determined by measuring the difference in the perpendicular distance from the inter-teardrop line to the superior aspect of the lesser trochanter of each hip [21,22] (Figure 1). Femoral offset of both hips was calculated as the distance from the center of the femoral head to a line bisecting the long axis of the femur (Figure 1). The center of the femoral head was identified by using the circle tool to match the femoral head diameter, which then provided the central point for the offset measurement. All measurements were performed by a single operator (AB) under the supervision of an arthroplasty surgeon (MT). The measurements were performed two times for each radiograph, and the means were used to compare the operated and non-operative control side within each group. Any difference in the leg length or femoral offset of 2 mm or more was considered as significant, while any difference of 5 mm or more was considered excessive since it results in un-physiological gait kinematics after hip arthroplasty [23].

The treating orthopedic surgeon was categorized by their subspecialty training and clinical practice. Group A comprised the surgeons who primarily performed hip arthroplasties (N = 3). Group B comprised all the non-arthroplasty surgeons (N = 10), including specialists in trauma (N = 3), sports medicine (N = 4), spine (N = 2) and oncology (N = 1). Of the 175 hips in the study, 52 hips were operated by surgeons in Group A and 123 hips by surgeons in Group B. Of the 123 hips in Group B, the subspecialty of the orthopedic surgeon performing the surgery was trauma in 77, sports medicine in 22, spine in 18 and oncology in 6 hips. There was no statistically significant difference in the demographics between the two groups. The mean age for patients in Group A was 76 ± 12 years (46 to 94) and in Group B it was 80 ± 11 years (43 to 102) (*p* = 0.21). Sixty percent of the patients in Group A were female compared to 67% in Group B. A cemented femoral stem was used in 61% of the cases in Group A compared to 60% cemented in Group B (*p* = 0.98). A Summit femoral stem (DePuy, Warsaw, IN) was used in 71% and 72% of the cases, while a VerSys LD/Fx stem (Zimmer, Warsaw, IN) was used in 29% and 28% of the cases in Groups A and B, respectively. The mean head size was 40 mm (range, 38 mm–56 mm) in Group A and 47 mm (range, 42 mm–57 mm) in Group B.

### Statistical Analysis

The Kolmogorov–Smirnov test was used to determine whether the data were normally distributed. For each hip, the difference in pre- and postoperative offset and leg length discrepancy (LLD) measurements were given as the difference between the means. Intra-observer reliabilities for all measurements were tested with intra-class-correlation coefficients (ICC) with a two-way random effects model for absolute agreement. A paired t-test was used to compare mean differences between Group A to Group B and Group A to each subspecialty (trauma, sports medicine, spine and oncology), regarding offset and leg length restoration. Statistical analyses were carried out using SPSS^®^ software (SPSS Inc, Chicago, IL, USA), and the level of significance was set at *p* < 0.05 for all statistical analyses.

## 3. Results

Overall, the leg lengths were restored to their preoperative value in 96% of the hips treated by the arthroplasty surgeons (Group A) and in 90% of those treated by the non-arthroplasty surgeons (Group B) (*p* = 0.25). The mean difference between the preoperative and postoperative leg lengths was 0.5 ± 2.6 mm in Group A and 0.5 ± 2.9 in Group B (*p* = 0.64). Over-lengthening of the leg by at least 5 mm occurred in 4% of the hips in Group A and in 6% of the hips in Group B (*p* = 0.73). Over-lengthening of the leg by 5 mm or more did not occur more frequently if the hip was done by an arthroplasty specialist than if it was done by a specialist in trauma (*p* = 0.90), sports medicine (*p* = 0.17), spine (*p* = 0.37) or oncology (*p* = 0.22) (Table 1). Shortening of the leg by at least 5 mm did not occur in Group A and occurred in 4% of the hips in Group B (*p* = 0.16). As compared to the hips done by the arthroplasty surgeons, shortening of the leg of at least 5 mm was more likely to occur if the HA was done by the spine surgeons (*p* = 0.02) or the oncology surgeons (*p* = 0.005), but there was no difference between the arthroplasty surgeons and the other subspecialties (Table 1). Overall, failure to correct the native leg lengths by either over- or under-lengthening the operated hip by 5 mm or more occurred more frequently with the trauma and spine subspecialists than with the arthroplasty surgeons (*p* = 0.002 and *p* = 0.01, respectively). No orthopedic subspecialty restored the native leg lengths better than the arthroplasty specialists.

Overall, the femoral offset was restored in 80% of the hips in Group A and in 63% of the hips in Group B (*p* = 0.031). The mean difference between the preoperative and postoperative femoral offset was −0.41 ± 6.6 mm in Group A and 3.2 ± 9.5 mm in Group B (*p* = 0.001). The offset was decreased by a least 5 mm in 16% of the hips in Group A and 15% in Group B (*p* = 0.93). However, an increase in offset of at least 5 mm more than the preoperative value was found in 16% of the hips in Group A and in 39% of the hips in Group B (*p* = 0.003). Although an overall change in femoral offset of at least 5 mm occurred more frequently in Group B than in Group A (55% versus 32%, *p* = 0.005), it did not occur significantly more often in any subspecialty group than in the arthroplasty group (Table 1). No orthopedic subspecialty restored the native femoral offset better than the arthroplasty specialists (Table 1).

Overall, the leg lengths and offset were both restored to within 2 mm of the native value in 29% of the hips in Group A and 7% of the hips in Group B (*p* < 0.001).

## 4. Discussion and Conclusions

This study addressed whether arthroplasty trained surgeons and non-arthroplasty orthopedic surgeons of varying subspecialties are uniformly capable of restoring the patient’s native leg length and femoral offset when performing an HA for a femoral neck fracture. Overall, failure to correct the native leg lengths by either over- or under-lengthening the operated hip by at least 5 mm occurred more frequently with the trauma and spine subspecialists than with the arthroplasty surgeons. Additionally, arthroplasty surgeons were more likely to restore the native femoral offset within 5 mm than non-arthroplasty surgeons but not significantly better than a surgeon of a particular subspecialty. No orthopedic subspecialty restored the native leg length or femoral offset better than the arthroplasty surgeons.

Our findings contrast with the only previous study comparing a group of arthroplasty and non-arthroplasty surgeons’ ability to restore hip biomechanics after HA [20]. In that study of 84 hemiarthroplasties, Lakstein et al. found better performance of the arthroplasty surgeons in restoring leg length but no difference in offset reconstruction. However, they did not specifically address which non-arthroplasty subspecialty performed differently than the arthroplasty surgeons and the number of outliers in each subspecialty. Contrary to these findings, this study only found that an excessive leg length discrepancy was more likely to occur if the HA was done by specific non-arthroplasty subspecialists. However, we found that non-anatomic correction of femoral offset occurred more frequently in in the non-arthroplasty group than with the arthroplasty group. The differences in the findings between the two studies can be related to the larger number of patients in this study; that the Lakstein et al. study eliminated half the patients who met the inclusion criteria, compared to 24% in this study; the differences in the femoral implants’ offset used in the two studies; and/or the surgeons’ expertise.

Generally, arthroplasty surgeons tend to strive for reestablishing or even moderately increasing femoral offset on the operated hip, as this results in a better range of motion and increased stability with a reduced risk of dislocation [9,24,25,26,27]. Likewise, arthroplasty surgeons have a tendency to maintain or over-lengthen the leg rather than err by shortening the leg in order to avoid dislocation. Therefore, it is not surprising that the arthroplasty group did not severely shorten the leg by 5 mm or more, while this occurred in 4% of the hips in the non-arthroplasty group. However, it was unexpected that the offset was decreased by at least 5 mm in 16% of the cases in Group A. Overall, 96% of the arthroplasty surgeons and 90% of the non-arthroplasty surgeons managed to restore or increase the leg length and offset less than 5 mm.

The findings in the study indicate that there is room for improvement, and it is important to make all orthopedic surgeons aware of this shortcoming so that they can preoperatively template these cases and ensure they have the appropriate implants to be able to restore the normal anatomy of the patient. Since it is currently recommended that the vast majority or all femoral neck fractures be treated with a cemented stem, an implant with varying femoral neck offsets should be considered in order to help restore the native hip biomechanics [28,29,30].

This study has several potential limitations. First, we utilized the contralateral femur to determine the native femoral offset and presumed that the patient’s leg lengths were equal prior to their fracture. Although this is not the case in all patients, it is commonly the case and is likely a reasonable assumption since the difference between both legs is normally clinically insignificant. Second, we do not know if the differences in leg lengths and offset between the two groups and subspecialists resulted in any clinical symptoms or complications. However, the intent of the study was to determine if there is a difference in the ability to restore the biomechanics of the hip by different specialists because of their training and bias, not to correlate this with their outcome. Finally, the same femoral implant with the same offset and neck length was not used in all cases, and this could have led to under- or overcorrecting the hip biomechanics by the varying surgeons.

Following a hemiarthroplasty for a displaced femoral neck fracture, the normal biomechanics of the hip are more likely to be restored by an arthroplasty-trained surgeon than by a non-arthroplasty trained surgeon. Although non-arthroplasty surgeons tended to restore leg lengths, they were less successful in restoring the femoral offset. Identifying the inconsistency of non-arthroplasty surgeons and, to a lesser degree, arthroplasty surgeons in restoring hip biomechanics during hemiarthroplasty for hip fractures is important for sensitizing surgeons to rectify this in the future with appropriate templating and femoral implant selection.

## Figures and Tables

**Table 1:** Statistical difference between the arthroplasty surgeons and the non-arthroplasty specialists.

	Trauma	Sport	Spine	Oncology
LLD ≥ 5 mm	0.002	0.02	0.55	0.05
LLD ≤ 5 mm	0.79	0.80	0.55	0.97
Offset ≥ 5 mm	0.90	0.17	0.37	0.22
Offset ≤ 5 mm	0.48	0.14	0.02	0.05

LLD: Leg length discrepancy.

## References

[1] Kannus P., Parkkari J., Sievanen H., Heinonen A., Vuori I., Jarvinen M. (1996). Epidemiology of hip fractures. Bone.

[2] Timperley A.J., Whitehouse S.L. (2009). Mitigating surgical risk in patients undergoing hip arthroplasty for fractures of the proximal femur. J. Bone Jt. Surg. Br..

[3] Olsen F., Kotyra M., Houltz E., Ricksten S.E. (2014). Bone cement implantation syndrome in cemented hemiarthroplasty for femoral neck fracture: Incidence, risk factors, and effect on outcome. Br. J. Anaesth..

[4] Wang Z., Bhattacharyya T. (2017). Outcomes of Hemiarthroplasty and Total Hip Arthroplasty for Femoral Neck Fracture: A Medicare Cohort Study. J. Orthop. Trauma.

[5] Rogmark C., Leonardsson O. (2016). Hip arthroplasty for the treatment of displaced fractures of the femoral neck in elderly patients. Bone Jt. J..

[6] Garvin K.L., Hartman C.W., Berry J.R., La D.J. (2017). Primary THA: Preoperative Planning and Templating. Advanced Reconstruction Hip 2.

[7] Asayama I., Chamnongkich S., Simpson K.J., Kinsey T.L., Mahoney O.M. (2005). Reconstructed hip joint position and abductor muscle strength after total hip arthroplasty. J. Arthroplast..

[8] Asayama I., Naito M., Fujisawa M., Kambe T. (2002). Relationship between radiographic measurements of reconstructed hip joint position and the Trendelenburg sign. J. Arthroplast..

[9] McGrory B.J., Morrey B.F., Cahalan T.D., An K.N., Cabanela M.E. (1995). Effect of femoral offset on range of motion and abductor muscle strength after total hip arthroplasty. J. Bone Jt. Surg. Br..

[10] Konyves A., Bannister G.C. (2005). The importance of leg length discrepancy after total hip arthroplasty. J. Bone Jt. Surg. Br..

[11] Charles M.N., Bourne R.B., Davey J.R., Greenwald A.S., Morrey B.F., Rorabeck C.H. (2005). Soft-tissue balancing of the hip: The role of femoral offset restoration. Instr. Course Lect..

[12] Rosler J., Perka C. (2000). The effect of anatomical positional relationships on kinetic parameters after total hip replacement. Int. Orthop..

[13] Friberg O. (1984). Biomechanical significance of the correct length of lower limb prostheses: A clinical and radiological study. Prosthet. Orthot. Int..

[14] Schmalzried T.P., Shepherd E.F., Dorey F.J., Jackson W.O., dela Rosa M., McKellop H.A., Amstutz H.C. (2000). Wear is a function of use, not time. Clin. Orthop. Relat. Res..

[15] Sakai T., Sugano N., Ohzono K., Nishii T., Haraguchi K., Yoshikawa H. (2002). Femoral anteversion, femoral offset, and abductor lever arm after total hip arthroplasty using a modular femoral neck system. J. Orthop. Sci..

[16] Witjes S., Smolders J.M., Beaule P.E., Pasker P., Van Susante J.L. (2009). Learning from the learning curve in total hip resurfacing: A radiographic analysis. Arch. Orthop. Trauma Surg..

[17] Treskes K., Voeten S.C., Tol M.C., Zuidema W.P., Vermeulen J., Goslings J.C., Toor E.A. (2017). Trauma surgery by general surgeons: Still an option for proximal femoral fractures?. Injury.

[18] Chiasson P.M., Roy P.D., Mitchell M.J., Chiasson A.M., Alexander D.I. (1997). Hip fracture surgery in Nova Scotia: A comparison of treatment provided by “generalist” general surgeons and orthopedic surgeons. Can. J. Surg..

[19] Girard J., Lavigne M., Vendittoli P.A., Roy A.G. (2006). Biomechanical reconstruction of the hip: A randomised study comparing total hip resurfacing and total hip arthroplasty. J. Bone Jt. Surg. Br..

[20] Lakstein D., Atoun E., Wissotzky O., Tan Z. (2017). Does restoration of leg length and femoral offset play a role in functional outcome one year after hip hemiarthroplasty?. Injury.

[21] Loughead J.M., Chesney D., Holland J.P., McCaskie A.W. (2005). Comparison of offset in Birmingham hip resurfacing and hybrid total hip arthroplasty. J. Bone Jt. Surg. Br..

[22] Ranawat C.S., Rao R.R., Rodriguez J.A., Bhende H.S. (2001). Correction of limb-length inequality during total hip arthroplasty. J. Arthroplast..

[23] Renenkawitz T., Weber T., Dullien S., Woerner M., Dendorfer S., Grifka J., Weber M. (2016). Leg length and offset differences above 5 mm after total hip arthroplasty are associated with altered gait kinematics. Gait Posture.

[24] Little N.J., Busch C.A., Gallagher J.A., Rorabeck C.H., Bourne R.B. (2009). Acetabular polyethylene wear and acetabular inclination and femoral offset. Clin. Orthop. Relat. Res..

[25] Bourne R.B., Rorabeck C.H. (2002). Soft tissue balancing: The hip. J. Arthroplast..

[26] Bicanic G., Delimar D., Delimar M., Pecina M. (2009). Influence of the acetabular cup position on hip load during arthroplasty in hip dysplasia. Int. Orthop..

[27] Massin P., Geais L., Astoin E., Simondi M., Lavaste F. (2000). The anatomic basis for the concept of lateralized femoral stems: A frontal plane radiographic study of the proximal femur. J. Arthroplast..

[28] Tanzer M., Graves S.E., Peng A., Shimmin A.J. (2018). Is Cemented or Cementless Femoral Stem Fixation More Durable in Patients Older than 75 Years of Age? A Comparison of the Best-performing Stems. Clin. Orthop. Relat. Res..

[29] Troelsen A., Malchau E., Sillesen N., Malchau H. (2013). A review of current fixation use and registry outcomes in total hip arthroplasty: The uncemented paradox. Clin. Orthop. Relat. Res..

[30] Taylor F., Wright M., Zhu M. (2012). Hemiarthroplasty of the hip with and without cement: A randomized clinical trial. J. Bone Jt. Surg. Am..

